# Curcumin Inhibits Proliferation and Epithelial-Mesenchymal Transition in Lens Epithelial Cells through Multiple Pathways

**DOI:** 10.1155/2020/6061894

**Published:** 2020-03-31

**Authors:** Huijun Liu, Yuxiang Mao, Bing Xia, Chongde Long, Xielan Kuang, Hao Huang, Jie Ning, Xinqi Ma, Han Zhang, Renchun Wang, Han Tang, Han Du, Jianhua Yan, Qingjiong Zhang, Xinyu Zhang, Huangxuan Shen

**Affiliations:** ^1^State Key Laboratory of Ophthalmology, Zhongshan Ophthalmic Center, Sun Yat-sen University, Guangzhou 510060, China; ^2^Hunan Cancer Hospital and the Affiliated Tumor Hospital of Xiang-Ya School of Medicine, Central South University, Changsha 410078, China; ^3^Biobank of Eye, State Key Laboratory of Ophthalmology, Zhongshan Ophthalmic Center, Sun Yat-sen University, Guangzhou 510060, China; ^4^The Second Clinical Medicine School of Lanzhou University, Lanzhou 730000, China

## Abstract

**Background:**

Posterior capsule opacification (PCO), a complication of extracapsular lens extraction surgery that causes visual impairment, is characterized by aberrant proliferation and epithelial-mesenchymal transition (EMT) of lens epithelial cells (LECs). Curcumin, exerting inhibitive effects on cell proliferation and EMT in cancer, serves as a possible antidote towards PCO.

**Methods:**

Cellular proliferation of LECs after treatment of curcumin was measured with MTT assay and flow cytometry. The transcriptional and expressional levels of proteins related to proliferation and EMT of LECs were quantified by western blotting and real-time PCR.

**Results:**

Curcumin was found to suppress the proliferation of LECs by inducing G_2_/M arrest via possible inhibition of cell cycle-related proteins including CDK1, cyclin B1, and CDC25C. It had also inactivated proliferation pathways involving ERK1/2 and Akt pathways in LECs. On the other hand, curcumin downregulated the EMT of LECs through blocking the TGF-*β*/Smad pathway and interfering Notch pathway which play important roles in PCO.

**Conclusions:**

This study shows that curcumin could suppress the proliferation and EMT in LECs, and it might be a potential therapeutic protection against visual loss induced by PCO.

## 1. Introduction

Cataract is a vision-impairing disease in which opacification and turbidness of the lens are formed. Various factors contribute to the pathological process of cataract, while the only solution is performing ophthalmic surgery extracting a muddy lens out of the capsule with or without the intraocular lens implantation. However, these surgeries are risky with complications. Posterior capsule opacification (PCO) is a common complication after cataract surgeries and causes setback of sight during postoperation period [1]. PCO occurs in 30% of adults undertaking the modern lens surgery [2], and 100% in children owning to their strong proliferation ability of lens epithelial cells (LECs). Additional treatments including lens capsule polishing, laser capsulotomy, posterior continuous curvilinear capsulorhexis, and anterior vitrectomy are only able to decrease the incidence by 15%, and they might increase the risk of postsurgical inflammation and other complications [3, 4]. Hirnschall showed a method of opening capsular bag that significantly reduced PCO in rabbits, but procedures in human eyes were not carried out [2]. A new surgical method of cataract removal in infants was reported lately, which had preserved endogenous LECs and achieved functional lens regeneration, avoiding the complication of PCO [5]. But for cataract in elder children and adults, effective prevention and therapy of PCO are still in huge demand.

Epithelial-mesenchymal transition EMT, a main characteristic process of PCO, includes the acquisition of mesenchymal cell-like morphology, diminished intercellular junctions, reduced expression of epithelial markers such as ZO-1 and E-cadherin, and accumulation of EMT markers including *α*-smooth muscle actin (*α*-SMA), fibronectin, and N-cadherin, in addition to the upregulated expression of transcription factors including Snail, Slug, ZEB, and Twist [1, 4]. Among different EMT inducers, transforming growth factor beta (TGF-*β*) is the best known. The TGF-*β* family causes biological reactions by mediating Smad transcription factors and regulates gene's expression. It also activates non-Smad pathways that exert nontranscriptional roles including epithelial junction dissolution, cytoskeletal reorganization, motility, and translational control. There are three isoforms of TGF-*β*, and they are all identified in humans. Among them, TGF-*β*2 is the predominant isoform in ocular media and the most important factor contributing to transdifferentiation and pathologic fibrosis of LECs [1, 4, 6, 7].

Curcumin as a component of turmeric (*Curcuma longa*), 6-heptadiene-3,5-dione,1,7-bis(4-hydroxy-3-methoxyphenyl)-1,(e,e)-1 with the CAS number: 458-37-7, has been medically used in Asian for centuries [8]. Curcumin is a natural polyphenol and recent researches have revealed its diverse biological functions including antioxidation, antifungus, antibacteria, antivirus, anti-inflammation, immune-modulation, antiproliferation, and tumor suppression [8–12]. Besides, curcumin plays a regulatory role in the metabolic diseases through hypoglycemic, lipid-lowering, and cardioprotection mechanisms [13–15]. Despite the universal functions, studies of curcumin were mostly focused on the antitumor effect in cancer therapy. One of the vital mechanisms of this effect was found to be inhibiting proliferation and EMT in cancers, where pleiotropic genetic effects and multiple cell signaling pathways participated [16–18]. Thus, it is highly possible that curcumin exhibits proliferation and EMT blocking effects on LECs, which leads to a potential solution of PCO.

In this study, we confirmed the abilities of curcumin inhibiting proliferation and TGF-*β*2-induced EMT of human LECs according to a dose-dependent manner involving multiple pathways. In addition to its various effects, curcumin was shown in our investigation to become a novel cure of cataract surgical PCO.

## 2. Materials and Methods

The study was conducted in accordance with the Basic and Clinical Pharmacology and Toxicology policy for experimental and clinical studies [19].

### 2.1. Cell Culture and Treatments

The LEC cell line SRA01/04 was cultured in Dulbecco's modified Eagle's medium (DMEM, Life Technologies, Auckland, New Zealand) containing 10% fetal bovine serum (Life Technologies, Auckland, New Zealand). Cells were grown in a humidified atmosphere containing 5% CO_2_ at 37°C and dissociated with 0.25% trypsin-0.04% EDTA solution. TGF-*β*2 was reconstituted with 4 mM HCl containing 0.1% BSA to a final concentration of 50 *μ*g/ml, and curcumin was dissolved in DMSO to a storage concentration of 1 mM. For the treatments with TGF-*β*2 or curcumin, cells were first seeded in 100 mm dishes, then treated with 10 ng/ml recombinant human TGF-*β*2 or various concentrations of curcumin for 48 h.

### 2.2. Cell Proliferation Assay

The proliferation of LECs under curcumin treatment was assessed with MTT assay. LECs were seeded into 96-well plates at a density of 4 × 10^3^ cells/well and grown overnight. Then, cells were treated with different doses (0 *μ*M, 5 *μ*M, 10 *μ*M, 20 *μ*M, and 40 *μ*M) of curcumin (Sigma-Aldrich, Louis, MO, USA) for 24 h, 48 h, and 72 h, respectively. The following procedures were conducted as described previously [20].

### 2.3. Cell Apoptosis and Cell Cycle Analysis

LECs treated with different concentrations of curcumin for 48 h were collected and washed with PBS; cell apoptosis and cell cycle were analyzed by flow cytometry (Millipore, Darmstadt, Hesse, Germany) with the MultiCaspase Kit and the Cell Cycle Kit (Millipore, Darmstadt, Hesse, Germany). All procedures were proceeded according to the manufacturer's protocols as we previously did [21, 22]. The statistical analysis was performed with the software version 1.3 of the Muse™ Cell Analyzer (Millipore, Darmstadt, Hesse, Germany).

### 2.4. Real-Time RT-PCR

Total RNA of cells treated with TGF-*β*2 or curcumin for 48 h was isolated with TRIzol reagent (Takara Bio, Kusatsu, Shiga, Japan). cDNA synthesis and real-time RT-PCR were underwent as previously described [20, 23]. Data collection and analysis were used with the additional application software (LightCycler 96, F. Hoffmann-La Roche Ltd.) version 1.1. And the relative quantitative analysis of mRNA was calculated by using the 2^−*ΔΔ*Cq^ method with normalization to GAPDH.

### 2.5. Western Blot Assay

Cell proteins were extracted with a lysis buffer solution containing phosphatase inhibitor from the LECs. The western blot procedures were referred to our previous work [20, 23]. GAPDH (Proteintech, Wuhan, Hubei, China) and tubulin alpha (Affinity Biotech, Kansas, MO, USA) were used as controls. Other antibodies used included CDK1, cyclin B1, CDC25C, E-cadherin, fibronectin, *α*-SMA (Boster, Wuhan, Hubei, China), Notch1, Notch2, Smad2/3, p-Smad2/3 (Santa Cruz, CA, USA), p38, p-P38, Akt, p-Akt, ERK1/2, and p-ERK1/2 (CST, MA, USA).

### 2.6. Statistical Analysis

Each experiment was performed in triplicate with same protocol. All data were expressed as mean ± S.E.M, and statistical analyses were processed with SPSS version 22.0 (SPSS Inc., Chicago, IL, USA). One-way analysis of variance was used to compare differences among groups. *P* < 0.05 is regarded as statistically significant.

## 3. Results

### 3.1. Curcumin Inhibited the Proliferation of LECs

**Figure 1:**
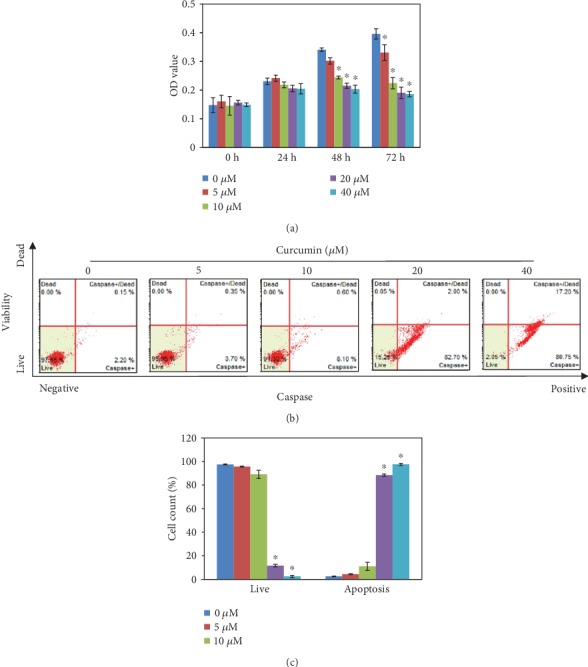
Curcumin treatments inhibited proliferation and increased apoptosis of LEC cells. (a) LECs were treated with curcumin (0, 5, 10, 20, and 40 *μ*M) for 72 h, and cell viabilities were assessed with an MTT assay in different time points. (b) LEC apoptosis was measured by flow cytometry using the MultiCaspase Kit after treatment with curcumin (0, 5, 10, 20, and 40 *μ*M) for 48 h. (c) A histogram of cell viability and cell apoptosis in (b) is shown. ^∗^*P* < 0.05 vs. cells treated without curcumin.

To investigate whether curcumin can prevent the aberrant proliferation of LECs, we used gradient concentrations of curcumin on SRA01/04 for 72 h. The MTT assay revealed that the proliferation of LECs was suppressed by curcumin in a time-dose-dependent manner (Figure 1(a)). Meanwhile, concentrations above 10 *μ*M had led to approximate OD values after the time point of 24 h, which suggested that the LEC cell growth arrest might be induced by 10^+^ *μ*M of curcumin *in vitro*. On the other hand, curcumin had induced apoptosis of LECs. Flow cytometry showed that curcumin had raised cell apoptosis to (88.4 ± 6.09) % and (97.5 ± 0.90) % at the concentration of 20 *μ*M and 40 *μ*M after 48 h, compared with the control group (2.53 ± 0.32) % (Figures 1(b) and 1(c)). However, the dosages of curcumin below 10 *μ*M did not affect cell viability significantly (*P* > 0.05).

### 3.2. Curcumin Inhibited Cell Cycle Progression of LECs at G_2_/M Phase

**Figure 2:**
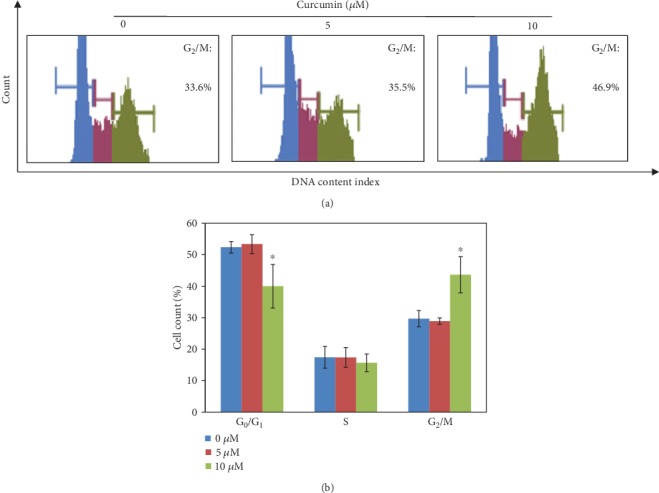
Curcumin induced cell cycle arrest in G2/M phase. (a) Cell cycle of LECs treated with curcumin (0, 5, and 10 *μ*M) were examined by flow cytometry. (b) A histogram of cell cycle in (a) is shown. ^∗^*P* < 0.05 vs. cells treated without curcumin.

**Figure 3:**
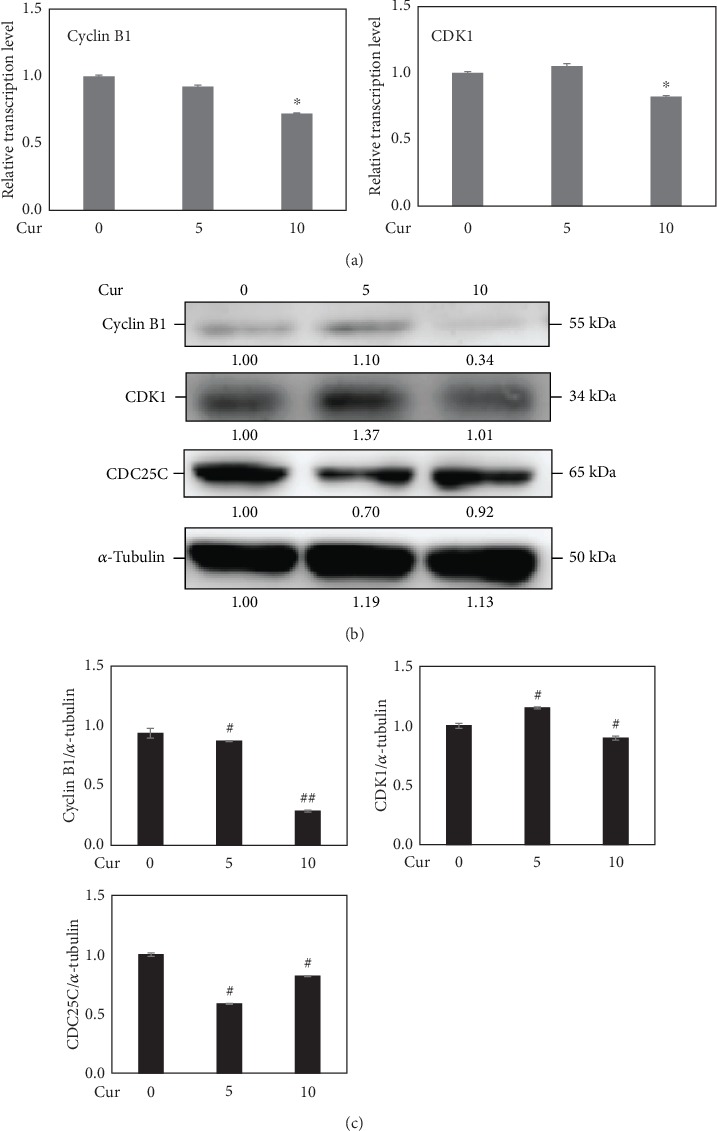
Curcumin decreased mRNA transcription and protein expression of cell cycle-related proteins at 10 *μ*M. (a) The mRNA transcriptions of cell cycle-related proteins cyclin B1 and CDK1 in LECs treated with curcumin (0, 5, and 10 *μ*M) for 48 h were detected by real-time PCR. GAPDH was used as an internal control. ^∗^*P* < 0.05, vs. the control. (b) Expressions of cell cycle-related proteins cyclin B1, CDK1, and CDC25C in curcumin-treated LECs were determined using western blot analysis. *α*-Tubulin was used as an internal control. (c) Densitometric analyses of the protein expression levels of cyclin B1, CDK1, and CDC25C from the western blots are shown. ^#^*P* < 0.05, ^##^*P* < 0.01 vs. the control.

Cell cycle was analyzed with flow cytometry after LECs were treated with curcumin for 48 h. The results showed that the curcumin of 10 *μ*M had increased cell numbers in the G_2_/M phase (43.6 ± 5.75)%, while 5 *μ*M curcumin did not affect the LEC cycle in the G_2_/M phase (28.9 ± 1.04)% (*P* > 0.05), compared with the control (29.7 ± 2.58)% (*P* < 0.05) (Figure 2). The cell cycle-regulating proteins of the G_2_/M phase were also examined. At the dose of 10 *μ*M, for 48 h, treatment of curcumin had downregulated the RNA transcriptional levels of *CDK1* and *cyclin B1*. Meanwhile, proteins including CDK1, cyclin B1, and CDC25C were inhibited significantly (Figure 3). But interestingly, curcumin of 5 *μ*M increased the protein expression of CDK1.

### 3.3. Curcumin Suppressed TGF-*β*2-Induced EMT of LECs

**Figure 4:**
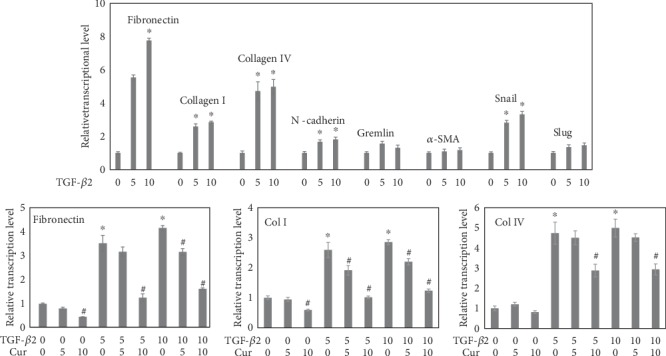
Curcumin inhibited the transcription of EMT-related genes induced by TGF-*β*2 in a dose-dependent manner. (a) Normalized mRNA levels of EMT-related genes (*fibronectin*, *collagen I*, *collagen IV*, etc.) in LECs treated with TGF-*β*2 (0, 5, and 10 ng/ml) for 24 h were detected by real-time PCR. (b) Normalized mRNA levels of EMT-related genes (*fibronectin*, *collagen I*, and *collagen IV*) in LECs treated with both TGF-*β*2 (0, 5, and 10 ng/ml) and curcumin (0, 5, and 10 *μ*M) for 24 h were detected by real-time PCR. ^∗^*P* < 0.05 vs. the control, ^#^*P* < 0.05 vs. cells without curcumin pretreatment.

**Figure 5:**
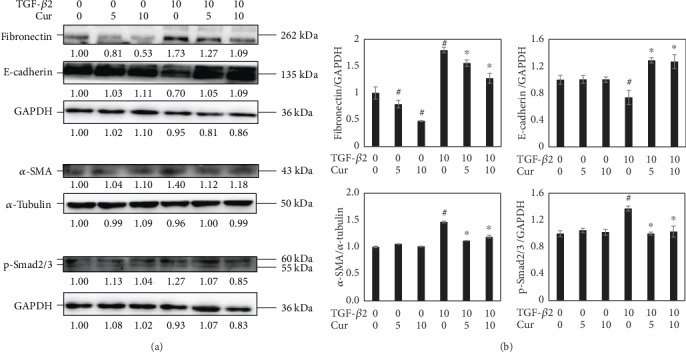
Curcumin suppressed the TGF-*β*2-induced EMT and TGF-*β*/Smad pathway activation. (a) EMT-related protein (fibronectin, *α*-SMA, and E-cadherin) and phosphorylated Smad2/3 in cells treated with TGF-*β*2 (0, 10 ng/ml) and curcumin (0, 5, and 10 *μ*M) for 24 h were detected by western blots. (b) Densitometric analyses of the protein expression levels of fibronectin, E-cadherin, *α*-SMA, and p-Smad2/3 from the western blots are shown. GAPDH and *α*-tubulin were used as internal controls. ^#^*P* < 0.05 vs. the control, ^∗^*P* < 0.05 vs. treatment with TGF-*β*2 only.

We had examined the RNA transcriptional levels of recombinant human TGF-*β*2-induced EMT level in LECs at the concentration of 10 ng/ml for 48 h. Firstly, gene expression markers of EMT including *fibronectin*, *collagen I*, *collagen IV*, and *N-cadherin*, and transcription factor *Snail* were all elevated correspondingly (Figure 4(a)). Secondly, in the LECs cocultured with curcumin of different concentrations (0 *μ*M, 5 *μ*M, and 10 *μ*M) and TGF-*β*2 of 10 ng/ml, the transcriptional levels of *fibronectin*, *collagen I*, and *collagen IV* were decreased in a dose-dependent manner (Figure 4(b)). By contrast, WB showed that the protein expressions of fibronectin and *α*-SMA were decreased, while E-cadherin was increased under cotreatment of curcumin and TGF-*β*2 (Figures 5(a) and 5(b)). Last but not the least, curcumin had inhibited the TGF-*β*2-induced phosphorylation of Smad2/3 and hence suppressed the TGF-*β*2 signaling pathway (Figure 5).

### 3.4. Curcumin and TGF-*β*2 Interacted with the Notch Signaling Pathway

**Figure 6:**
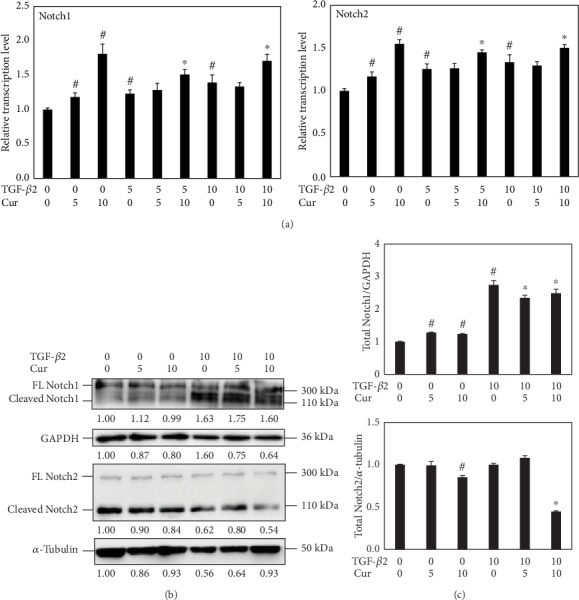
TGF-*β*2 and curcumin activated the transcription of *Notch1* and *Notch2*, while curcumin decreased the expression of Notch1 and Notch2 when coincubated with TGF-*β*2 in a dose-dependent way. (a) Normalized mRNA levels of *Notch1* and *Notch2* in cells treated with TGF-*β*2 and curcumin for 48 h were examined by real-time PCR. (b) Protein levels of Notch1 and Notch2 with TGF-*β*2 and curcumin treatments for 48 h were detected by western blots. (c) Densitometric analyses of the protein expression levels of Notch1 and Notch2 from the western blots are shown. GAPDH and tubulin were used as internal controls. ^#^*P* < 0.05 vs. the control, ^∗^*P* < 0.05 vs. treatment with TGF-*β*2 only.

The transcriptional level of *Notch1* and *Notch2* RNAs elevated in LECs treated with curcumin, TGF-*β*2, or both. However, the expressions of Notch were shown to be reducing in LECs with cotreatment of TGF-*β*2 and curcumin (Figure 6).

### 3.5. Curcumin Blocked the ERK1/2 and Akt Signaling Pathway

**Figure 7:**
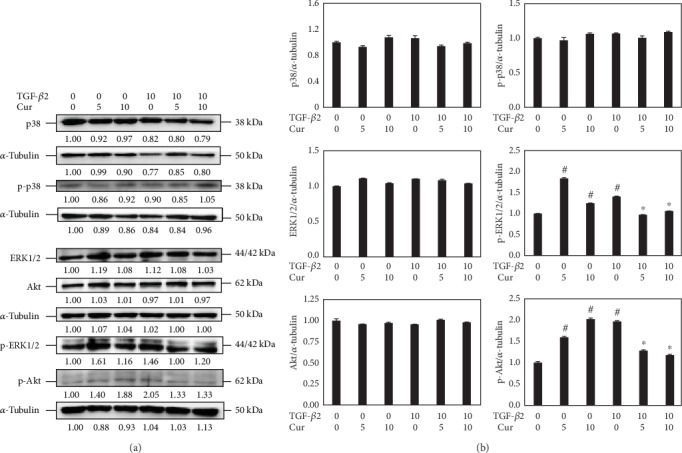
Curcumin suppressed TGF-*β*2-induced activation of the ERK1/2 and Akt pathway while the p38 pathway was not affected. (a) Expressions of p38, ERK1/2, Akt, and their phosphorylated forms in LECs treated with TGF-*β*2 (0, 10 ng/ml) and curcumin (0, 5, and 10 *μ*M) for 48 h were detected by western blots. (b) Densitometric analyses of the protein expression levels of p38, p-p38, ERK1/2, p-ERK1/2, Akt, and p-Akt from the western blots are shown. GAPDH and tubulin were used as internal controls. ^#^*P* < 0.05 vs. the control, ^∗^*P* < 0.05 vs. treatment with TGF-*β*2 only.

Both the ERK1/2 pathway and Akt signaling pathway are connected with proliferation and cell growth [4]. WB showed that curcumin had decreased phosphorylation of ERK1/2 and Akt induced by TGF-*β*2. In this way, curcumin was capable of blocking the ERK1/2 and Akt signaling pathways; thus, it is able to regulate cell proliferation and growth. However, p38 and p-P38 of the MAPK pathway were not changed during TGF-*β*2 and curcumin treatments (Figures 7(a) and 7(b)).

## 4. Discussion

This study has revealed the potential value of curcumin in PCO treatment for the first time. PCO is one of the most common complications after cataract surgery, which manifests visual loss resulting from proliferation and EMT of LECs. Despite posterior capsulotomy by using laser or surgery being accessible for PCO treatment, they will increase the medical expenses, suffering of patients, and possible risks of complications. From the fact that more patients are receiving extracapsular lens extraction surgery worldwide, PCO has developed into a social burden. Therefore, discovering novel and brief therapeutic methods is urgently required. Curcumin, a component extracted from spice turmeric in the first place, presented abilities of suppressing cellular proliferation and inducing apoptosis in cancers [16, 24, 25]. In this research, we studied the functional mechanism of curcumin on the proliferation and EMT of human LEC cell line SRA01/04 and revealed its valuable potential in solving PCO.

Following the experiments on cellular proliferations, the OD values of LECs were suppressed by curcumin according to a time-dose manner (Figure 1(a)). And at doses under 10 *μ*M, curcumin showed no signs of increasing apoptosis (Figures 1(b) and 1(c)), so further studies were performed with curcumin of 0, 5, and 10 *μ*M. Experiments afterwards had also shown inhibiting effects of curcumin on LEC proliferation. The G_2_/M phase arrest uncovered the possible time point where proliferating was stopped (Figure 2). In the process of cell cycle [26–29], firstly, cyclin B1 is an important protein required in G_2_/M transition; secondly, phosphorylation of Thr14, Tyr15, and Thr161 in CDK1 by kinases upstream such as Myt1 and WEE activate the CDK1-cyclin B1 complex that regulates phase transition from G_2_ to M; thirdly, CDC25C activates the complex through dephosphorylating Thr14/Tyr15 and then initiates the process of mitosis. The downregulation of these cell cycle time point proteins resulted from curcumin treatment explaining the molecular mechanism of G_2_/M phase arrest in LECs (Figure 3). As for curcumin of 5 *μ*M, it increased the expression of CDK1; it is reported by Chen et al. that a low dose of curcumin (5 *μ*M) inhibits proliferation of colorectal cancer cells by inducing senescence rather than apoptosis [30], and besides, Müllers et al. showed the activity of CDK1/2 after DNA damage promotes senescence [31], so the low dose of curcumin may lead to senescence via CDK1 presumably. On the other hand, it was reported that TGF-*β*2 could activate the Akt pathway in human subconjunctival fibroblasts, and Akt along with the MAPK pathway contributed to TGF-*β*2-induced upregulation of Jagged-1 in retinal epithelial (RPE) cells [32, 33]. We showed that the mechanism of growth arrest caused by curcumin in LECs under TGF-*β* treatment was related to ERK1/2 blockage and Akt inhibition but not p38 (Figure 7). As all is presented, curcumin is able to inhibit the cellular proliferations of LECs and shows a possible capability in controlling cell growth in PCO.

TGF-*β* is an effective EMT inducer in both normal and pathological conditions [7] and a promoter of mesenchymal transitions in cancer cells [34]. In a previous research, TGF-*β* was also demonstrated to induce EMT in lens, which increased the possibility of PCO onset [1]. In this study, 10 ng/ml of TGF-*β*2 successfully induced EMT in LECs by elevating protein markers including fibronectin, collagen I, and collagen IV. After treatment of curcumin, the RNA transcriptional levels of the markers *fibronectin*, *collagen I*, and *collagen IV* were downregulated (Figure 4). Correspondingly, the protein levels of fibronectin and *α*-SMA were decreased (Figure 5). However, E-cadherin was found to be upregulated by curcumin (Figure 5). This can be explained that curcumin abrogated the mesenchymal feature induced by TGF-*β*2 and restored the epithelial feature of LECs. Research showed that baicalin exerts antifibrosis effects via blocking the TGF-*β*/Smad signaling pathway [35]. Thus, the TGF-*β*/Smad signaling pathway is a potential target of alleviating pathogenic-fibrosis processes. In this study, curcumin had downregulated the level of p-Smad2/3 (Figure 5). It is known that the TGF-*β* signaling pathway is activated by Smad2/3 phosphorylation, causing various processes such as EMT and organ fibrosis [1]. When binding to the receptor, TGF-*β* activates the TGF-*β*/Smad signaling pathway and regulates the expression of target genes [36]. Hence, curcumin abrogated EMT by inactivating the p-Smad2/3 in TGF-*β*2-treated LECs. The Notch pathway was also involved in the process. It was reported that *Notch* acts as an oncogene and also as a tumor suppressor depending on the context, which is playing an important role in TGF-*β*2-induced EMT [37, 38]. Further researches had shown that the Jagged/Notch pathway is necessary for EMT induced by TGF-*β* in RPE, while there is a complex interplay existing between ERK1/2, TGF-*β*/Smad, and Jagged/Notch pathways in EMT regulation [4, 33, 39]. Although the transcriptional level and translational level of Notch1 and Notch2 seemed to be in different patterns as shown in the results (Figure 6), it highlighted the possible role of posttranscriptional modulation. And from the protein level, we found that curcumin attenuated the increase of Notch1 and 2 with TGF-*β* incubation in a dose-dependent manner which may shed light on researches in the future. Put together, the process of EMT in LECs was attenuated by curcumin via various accesses involving TGF-*β*/Smad and Notch signaling pathways.

Recently, a lot of researches have focused on the mechanism of curcumin's inhibition of malignant tumor cells as well as its function on noncoding RNAs which play an important role in the oncogenesis via the transcriptional and posttranscriptional levels. The main noncoding RNAs in this process include microRNA (~22 nts) and long noncoding RNA (>200 nts), and they can both act as tumor suppressors or tumor oncogenes in tumorigenesis [40]. Oncogene lncRNA HOTAIR is upregulated in numerous tumors and may be involved in curcumin-induced inhibition of renal carcinoma cell metastasis [41]. In colorectal cancer, curcumin regulates the tumor suppressor GAS5 to modulate the metastatic pathways [42]. It is also showed that curcumin produces repression of miRNA-21 expression, and PTEN the downstream target gene is markedly increased in A549 cells upon incubation with curcumin, finally leading to a marked inhibition of cell growth and inducing apoptosis [43]. Considering noncoding RNAs' involvement in the inhibition of proliferation and metastasis in cancer cells under curcumin incubation, we may speculate that noncoding RNAs could also be regulated by curcumin in the proliferation and EMT of LECs, and further investigation will be needed in the future.

In conclusion, this study has revealed the potential value of curcumin in PCO treatment for the first time. It has not only provided evidence that curcumin could suppress LECs proliferation by G_2_/M cell cycle arrest and blocking ERK1/2 along with Akt pathway but also showed that the curcumin had inhibited TGF-*β*-induced EMT through suppressing the TGF-*β*/Smad pathway and interfered with the Notch pathway. Though there are still works to be done to further understand the effects and functional mechanisms of curcumin on cellular pathology, our work has illustrated its possibly curative effect in PCO.

## Data Availability

In this manuscript 6061894 titled “Curcumin Inhibits Proliferation and Epithelial-mesenchymal Transition in Lens Epithelial Cells through Multiple Pathways” submitted to BioMed Research International, the nature of the data is figures in the manuscript. So everyone can access them when reading without any restrictions on data access.
